# Development and validation of AI-derived segmentation of four-chamber cine cardiac magnetic resonance

**DOI:** 10.1186/s41747-024-00477-7

**Published:** 2024-07-12

**Authors:** Hosamadin Assadi, Samer Alabed, Rui Li, Gareth Matthews, Kavita Karunasaagarar, Bahman Kasmai, Sunil Nair, Zia Mehmood, Ciaran Grafton-Clarke, Peter P. Swoboda, Andrew J. Swift, John P. Greenwood, Vassilios S. Vassiliou, Sven Plein, Rob J. van der Geest, Pankaj Garg

**Affiliations:** 1 Department of Cardiovascular and Metabolic Health, Norwich Medical School, University of East Anglia, Norwich, Norfolk, UK; 2 Norfolk and Norwich University Hospitals NHS Foundation Trust, Norwich, Norfolk, UK; 3 Department of Infection, Immunity & Cardiovascular Disease, University of Sheffield, Sheffield, UK; 4 Division of Biomedical Imaging, Leeds Institute of Cardiovascular and Metabolic Medicine, University of Leeds, Leeds, UK; 5 Department of Radiology, Division of Image Processing, Leiden University Medical Center, Leiden, The Netherlands

**Keywords:** Artificial intelligence, Deep learning, Heart diseases, Magnetic resonance imaging (cine), Prognosis

## Abstract

**Background:**

Cardiac magnetic resonance (CMR) in the four-chamber plane offers comprehensive insight into the volumetrics of the heart. We aimed to develop an artificial intelligence (AI) model of time-resolved segmentation using the four-chamber cine.

**Methods:**

A fully automated deep learning algorithm was trained using retrospective multicentre and multivendor data of 814 subjects. Validation, reproducibility, and mortality prediction were evaluated on an independent cohort of 101 subjects.

**Results:**

The mean age of the validation cohort was 54 years, and 66 (65%) were males. Left and right heart parameters demonstrated strong correlations between automated and manual analysis, with a *ρ* of 0.91−0.98 and 0.89−0.98, respectively, with minimal bias. All AI four-chamber volumetrics in repeatability analysis demonstrated high correlation (*ρ* = 0.99−1.00) and no bias. Automated four-chamber analysis underestimated both left ventricular (LV) and right ventricular (RV) volumes compared to ground-truth short-axis cine analysis. Two correction factors for LV and RV four-chamber analysis were proposed based on systematic bias. After applying the correction factors, a strong correlation and minimal bias for LV volumetrics were observed. During a mean follow-up period of 6.75 years, 16 patients died. On stepwise multivariable analysis, left atrial ejection fraction demonstrated an independent association with death in both manual (hazard ratio (HR) = 0.96, *p* = 0.003) and AI analyses (HR = 0.96, *p* < 0.001).

**Conclusion:**

Fully automated four-chamber CMR is feasible, reproducible, and has the same real-world prognostic value as manual analysis. LV volumes by four-chamber segmentation were comparable to short-axis volumetric assessment.

**Trials registration:**

ClinicalTrials.gov: NCT05114785.

**Relevance statement:**

Integrating fully automated AI in CMR promises to revolutionise clinical cardiac assessment, offering efficient, accurate, and prognostically valuable insights for improved patient care and outcomes.

**Key points:**

• Four-chamber cine sequences remain one of the most informative acquisitions in CMR examination.

• This deep learning-based, time-resolved, fully automated four-chamber volumetric, functional, and deformation analysis solution.

• LV and RV were underestimated by four-chamber analysis compared to ground truth short-axis segmentation.

• Correction bias for both LV and RV volumes by four-chamber segmentation, minimises the systematic bias.

**Graphical Abstract:**

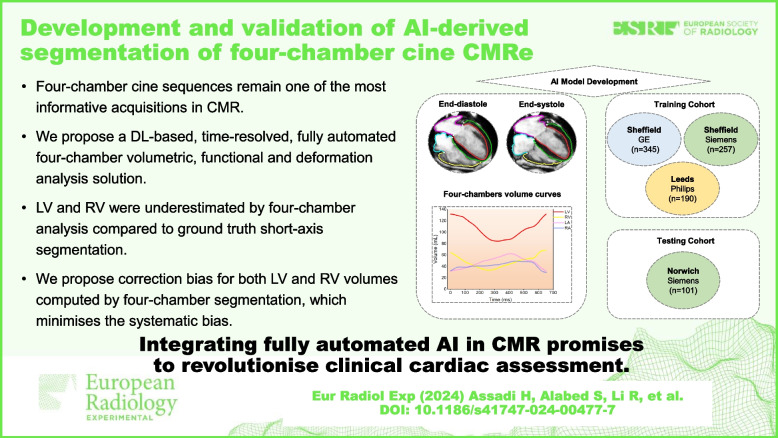

**Supplementary Information:**

The online version contains supplementary material available at 10.1186/s41747-024-00477-7.

## Background

Cardiac magnetic resonance (CMR) is an established imaging modality for cardiovascular assessment [1]. CMR offers one of the best spatial resolutions for volumetric assessment of the heart. In routine practice, a stack of cine images acquired in the left ventricle (LV) short-axis orientation is the reference method for LV and right ventricle (RV) volumetric assessment, and several software solutions offer deep learning artificial intelligence (AI) segmentation models for short-axis cine volumetric assessment [2–8]. Cine images in the four-chamber long-axis plane are also routinely acquired in most CMR studies [9]. Analysis based on the four-chamber may be useful in cases where the short axis has misregistration or temporal issues due to undersampling, or it may provide a complimentary analysis to the short-axis stack for internal validation. Furthermore, assessment of the longitudinal function of the heart is typically performed using a long-axis view per the Society for Cardiovascular Magnetic Resonance recommendations [10]. The four-chamber view provides rapid analysis that is often comparable to the results obtained through transthoracic echocardiography [11].

Automated heart segmentation using the four-chamber cine has previously mostly focused on a single chamber, commonly the LV [3, 12], the left atrium (LA) [3, 13] or the right atrium (RA) [8]. Moreover, commercially available CMR analysis software, CVi42 (Circle Cardiovascular Imaging Inc., Calgary, Canada; https://www.circlecvi.com/), can automatically analyse LV, LA, and RA on all cardiac phases from two orthogonal planes but not the RV. Other commercially available CMR software solutions, including Medis Suite MRI (Medis medical imaging systems, Leiden, The Netherlands; https://www.medisimaging.com/), HeartVista (HeartVista Inc., CA, USA; https://www.vista.ai/), and Caas MR solutions (Pie Medical Imaging, Maastricht, The Netherlands; https://www.piemedicalimaging.com/), have integrated AI segmentation tools for the LV and RV but not for all four chambers in the long-axis view. A comprehensive, time-resolved four-chamber cine segmentation of all four chambers will allow for the assessment of not only the global longitudinal function but also the inference of LA and RA function [14]. Additionally, as the evaluation is being made in one cine sequence, issues with coregistration are less likely.

The main objective of this study was to train and develop a deep learning AI model of time-resolved segmentation of all heart chambers using the four-chamber cine sequence in a multicentre and multivendor dataset. The second objective was to validate the agreement of the AI model externally with manual four-chamber and automated short-axis segmentation analyses. The third objective was to investigate the prognostic value of the AI model in the validation cohort.

## Methods

### Study cohort

This multicentre, multivendor, and retrospective observational study consisted of 814 previously prospectively recruited individuals between 2014 and 2018 who were selected for training, comprising studies of 624 patients in the ASPIRE registry from Sheffield Teaching Hospital (367 scans with GE Healthcare equipment and 257 scans Siemens Healthineers equipment), as well as 190 subjects from Leeds Teaching Hospitals NHS Trust (with Philips Healthcare equipment) [15–17]. To assess the performance of the model, a separate population comprising 101 prospectively recruited patients (studied with Siemens Healthineers equipment) from the PREFER-CMR registry in Norfolk and Norwich University Hospitals was used. The inclusion criteria specific to this study for the derivation and validation cohorts were individuals over the age of 18 with a clinical indication for CMR, good quality scan for segmentation and who provided written informed consent. The exclusion criteria for all subjects were body weight > 120 kg, inability to lie flat, pregnancy, incompatible devices or implants or any other contraindication to CMR, including allergy to contrast, claustrophobia, and end-stage renal impairment (estimated glomerular filtration rate < 30 mL/min).

### Ethics approval and consent to participate

The research adhered to the guidelines outlined in the 2013 version of the Declaration of Helsinki. Data acquisition and handling were authorised by the National Research Ethics Service in the UK, with approval number 21/NE/0149. A pragmatic opt-out informed consent was obtained from all subjects included in the study [18, 19].

### CMR protocol

CMR images of the training cohort for this study were acquired on scanners from three vendors:

1. 1.5-T HDx system by GE Healthcare (Chicago, IL, USA); the CMR protocol included baseline survey images and standard cine images with 8-mm slice thickness and 20 phases per cardiac cycle, repetition time of 3.7 ms and echo time of 1.6 ms using a cardiac-gated balanced steady-state free precession (bSSFP) sequence;
2. 1.5-T Avanto system by Siemens Healthineers (Erlangen, Germany); the CMR protocol included baseline survey images and standard cine images, with 6-mm slice thickness and 25 phases per cardiac cycle, repetition time of 38.92 ms and echo time of 1.13 ms using a cardiac-gated bSSFP sequence;
3. 1.5-T Ingenia system by Philips Healthcare (Best, the Netherlands); the CMR protocol included baseline survey images and standard cines with 8-mm slice thickness, 30 phases per cycle, repetition time of 2.72 ms and echo time of 1.36 ms using a bSSFP single-slice breath-hold sequence.

The validation cohort consisted solely of studies performed with a 1.5-T Magnetom Sola system (Siemens Healthineers, Erlangen, Germany) acquired in a clinical setting, not used in training the model. Cine CMR acquisitions were performed using a bSSFP sequence. The CMR protocol included baseline survey images and cine sequences. Following planning sequences, four-chamber cine images were acquired, followed by a stack of short-axis cine images covering apex to base. Standard cine images were obtained with 8-mm slice thickness, 30 phases per cycle, repetition time of 2.71 ms and echo time of 1.13 ms, field of view 360 × 289.3 mm^2^ with phase 80.4%, number of signal averages 1, matrix 224 × 180 (phase), bandwidth 167.4 kHz (930 Hz/Px), flip angle 80°, and GRAPPA acceleration with a factor of 2.

### CMR manual image analysis

Endocardial contours for the LV, RV, LA, and RA were manually drawn in all cardiac phases using point-by-point tracing on the four-chamber cine. Papillary muscles and trabeculations were included in the volume calculation [10, 20]. Epicardial contours in all cardiac phases were also drawn for the LV. All image analyses used the MASS research software (MASS, Version 2023-EXP, Leiden University Medical Center, Leiden, The Netherlands). Manual annotations for the GE and Philips scans in the training cohort were previously described [15]. For the Siemens cases in the training and testing datasets, manual annotations were performed by H.A. (four years of advanced CMR experience). All manual annotations were performed without the observers’ knowledge of the patient’s clinical details. P.G. (European Association of Cardiovascular Imaging level-III certified expert with over 10 years of CMR experience) reviewed all the manual contours to ensure accuracy.

### AI model training

In this work, we used a fully convolutional neural network previously developed for automated segmentation of four-chamber cine CMR [15]. This study showed high agreement between automated and manually derived RA area measurements, and the correlation between AI-derived RA areas and invasive haemodynamics was demonstrated. The original model was trained on a combination of GE (*n* = 367) and Philips (*n* = 80) scan data (total *n* = 447), which were randomly selected from the ASPIRE in Sheffield [21] and Leeds registries [16, 17]. To improve and increase the model ability to be applied broadly, 257 Siemens and 110 Philips scans with manually traced contours were added to the training set. The final model training set comprised 367 GE, 190 Philips, and 257 Siemens scans from three centres.

The CNN used in this study had a similar architecture to U-NET, consisting of 16 convolutional layers incorporating residual learning units. The implementation was carried out using Python (https://www.python.org/) and TensorFlow (https://www.tensorflow.org/). Images were resampled to a pixel spacing of 1 mm and cropped to a matrix size of 256 × 256 pixels, using zero filling as required. During training, data augmentation was performed on the images by randomly rotating, flipping, shifting, and modifying their intensities, which created new training samples. A total of 814 manually annotated four-chamber cine series were used for training, corresponding to 18,289 images. The Adam optimiser method was used for training, and the learning rate was set at 0.001. The loss function used was cross-entropy. Each training batch comprised a random selection of 20 images, and the number of epochs was set to 50. The CNN raw output was a labelled image with six possible label values corresponding to one of the four cardiac chambers, the background, or the left ventricular myocardium. The largest connected component was extracted for each cardiac label, and a closed and spatially smoothed contour was generated around the extracted region for each cardiac label. The area of the cardiac cavities was then calculated as the area surrounded by the generated contours.

**Fig. 1:**
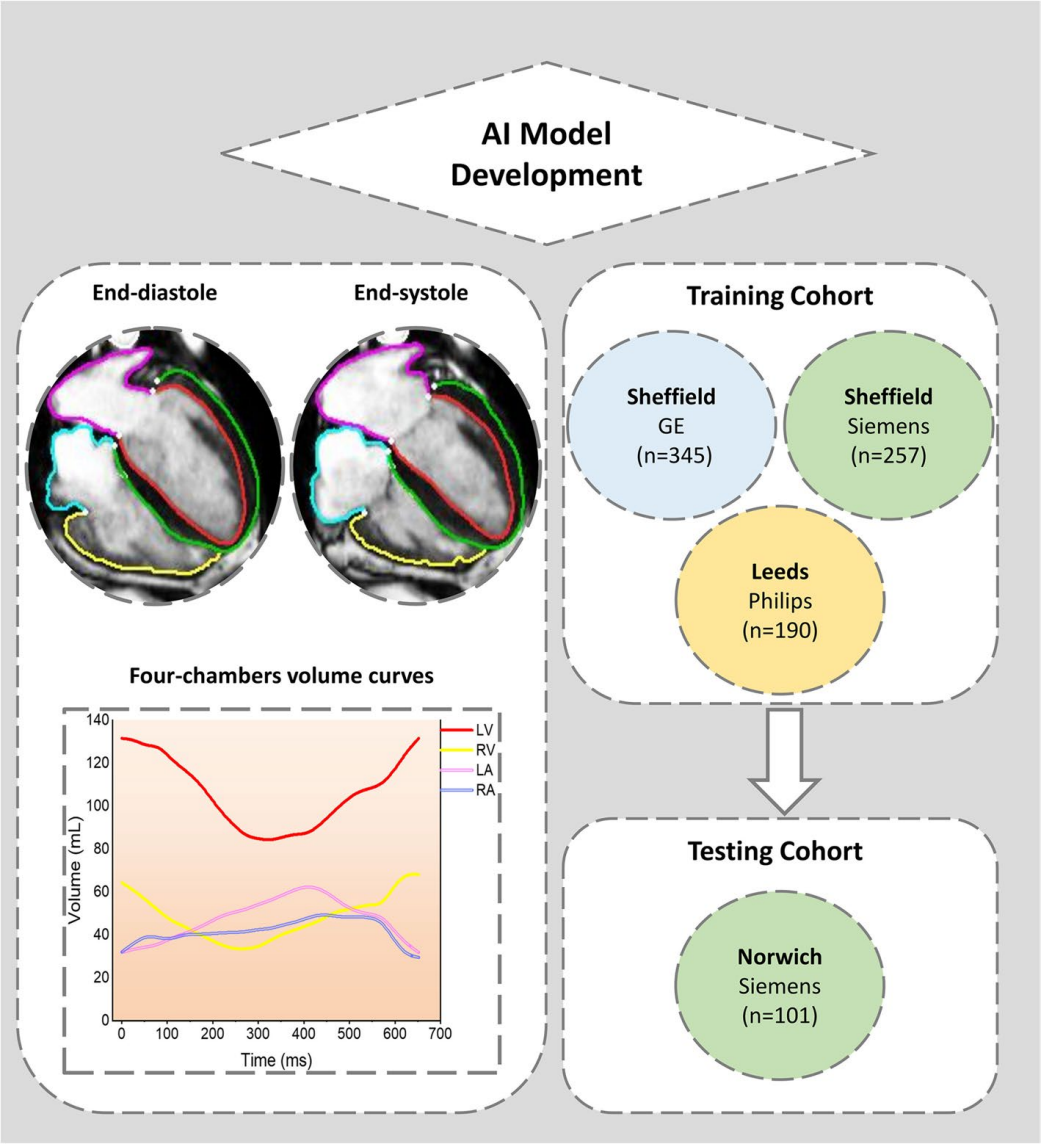
Overview of study flow chart. *AI* Artificial intelligence, *LA* Left atrium, *LV* Left ventricle, *RA* Right atrium, *RV* Right ventricle

For quality control, the AI-generated segmentations and time-resolved volume curves throughout all cardiac phases for all four chambers were evaluated by H.A. A visual assessment-based scoring system of satisfactory, suboptimal, or failure was used. Satisfactory categorisation comprised instances of perfect annotations or minor errors deemed insignificant to affect the time-resolved volume curves. Suboptimal categorisation comprised annotations with errors significant enough to impact time-resolved volume curves. Failure was characterised by either missing annotations or substantial failure in contouring cardiac chambers (Supplemental Video S1). The image acquisition quality was also assessed for artefacts and slice position errors. The experimentations were conducted on a regular computer featuring an Intel Core i7 CPU, 64 GB of internal RAM, and an Nvidia GTX 1080 TI GPU with 12 GB of memory. A central illustration demonstrating an overview of the study flow chart is shown in Fig. 1.

### Statistical analysis

Normal distribution was tested using the Shapiro–Wilk test (Supplemental Table S1). Continuous variables were summarised using the mean value and standard deviation (SD) or median with interquartile range to provide an overview of central tendency and data dispersion. Categorical data were expressed as frequencies and percentages. To compare continuous variables, we used a two-sample independent *t*-test. Correlations between the segmentation methods were evaluated using Pearson coefficient of rank correlation (*r*) for normally distributed data or Spearman coefficient of rank correlation (*ρ*) for non-parametric data. We used Bland–Altman plot analysis to check for agreement and bias between the methods. We calculated the within-subject coefficient of variation (CoV) as SD of the differences divided by the mean. We used the Cox proportional hazard model and the Kaplan–Meier analysis for univariable and multivariable prognosis analysis. Statistical analyses were conducted using SPSS Statistics version 29 (IBM, Chicago, USA) and confirmed in MedCalc version 22.009 (MedCalc Software, Ostend, Belgium). Unless otherwise indicated, all statistical tests were two-tailed, and significance was defined as a *p*-value < 0.05.

## Results

### Study* population*

The study sample included 915 subjects, of which 814 were used to train the model. The training dataset included CMR studies from two centres and three vendors (Sheffield: 367 GE scans and 257 Siemens scans; Leeds: 190 Philips scans). Demographic data for the derivation cohorts were previously described [8, 15, 16, 22]. To validate our model externally, we used CMR data from one centre and one vendor (Norwich: 101 Siemens scans). The demographic data of the validation cohort are shown in Table 1. The mean age was 54 years, and 65% of the cohort were male.

**Table 1:** Study demographics of the external validation cohort

Demographics	All (*n* = 101)	Alive (*n* = 85)	Dead (*n* = 16)	*p*-value
Age, years	54.2 ± 16.5	51.6 ± 16.3	67.8 ± 9.7	** < 0.001**
Male sex, *n* (%)	66 (65)	51 (60)	15 (94)	**0.009**
Body surface area, m^2^	1.97 ± 0.21	1.95 ± 0.21	2.05 ± 0.20	0.095
Smokers, *n* (%)	33 (33)	28 (33)	5 (31)	0.871
Hypertension, *n* (%)	31 (31)	24 (28)	7 (44)	0.222
Diabetes mellitus, *n* (%)	10 (10)	7 (8)	3 (19)	0.201
Atrial fibrillation, *n* (%)	18 (18)	13 (15)	5 (31)	0.131
Ischaemic heart disease, *n* (%)	30 (30)	21 (25)	9 (56)	**0.011**
Myocardial infarction, *n* (%)	4 (4)	4 (5)	16 (100)	0.381
Chronic obstructive pulmonary disease, *n* (%)	5 (5)	4 (5)	1 (6)	0.791
Oedema, *n* (%)	10 (10)	6 (7)	4 (25)	**0.030**
New York Heart Association functional class, *n* (%)	I, 74 (73)	I, 63 (74)	I, 11 (69)	0.901
	II, 12 (12)	II, 9 (11)	II, 3 (19)	
	III, 15 (15)	III, 13 (15)	III, 2 (13)	
Haemoglobin, g/dL	130.1 ± 40.6	126.7 ± 43	146.7 ± 18	0.073
Creatinine, μmol/L	84.3 ± 23.9	81.9 ± 23.3	96.3 ± 23.8	**0.027**
Urea, mmol/L	7.2 ± 8.6	7.2 ± 9.4	7.1 ± 2.5	0.992

Data are given as mean ± standard deviation

In the validation cohort, follow-up data between undergoing CMR and the pre-specified end date were obtained from the hospital clinical notes. During a mean follow-up period of 6.75 years, 16 (16%) of patients died. Patients who died were older (68 ± 10 *versus* 52 ± 16 years, *p* < 0.001), and more males died than females (15 *versus* 1, *p* = 0.009). There were no statistical differences between patients who died and those who survived in terms of comorbid history (*p* > 0.05). All patients who reached the endpoint had a history of myocardial infarction. One-third of patients who died were smokers and or had a history of atrial fibrillation (5 of 16). There was a high prevalence of ischaemic heart disease (56%), followed by hypertension (44%). Nearly a quarter of the patients were diabetics, and a substantial minority had either a history of coronary revascularisation procedure (19%) or chronic obstructive pulmonary disease (6%).

### CMR evaluation

CMR characteristics of the validation cohort are demonstrated in Table 2. AI-derived segmentation yielded higher median values than manual analysis for LA end-diastolic volume (EDV) (90 mL *versus* 86 mL), LA stroke volume (SV) (51 mL *versus* 46 mL), LV EDV (156 mL *versus* 155 mL), LV SV (91 mL *versus* 86 mL), LV peak filling rate (487 mL/s *versus* 448 mL/s), RA SV (33 mL *versus* 31 mL), RV EDV (84 mL *versus* 76 mL), and RV SV (54 mL *versus* 50 mL). AI-derived segmentation overestimated LV and RV cardiac outputs (median (interquartile range) 5,878 (5,046–7,178) mL/min *versus* 5,506 (4,888–6,910) mL/min, and 3,608 (2,671–4,336) mL/min *versus* 3,331 (2,505–4,158) mL/min, respectively). While LA, LV and RA ejection fraction (EF) measurements were lower in the manual segmentation method, RV EF was slightly lower in the automated approach (66% *versus* 68%).

**Table 2:** Results of manual and automated analyses

	**Manual segmentation**	**Automated segmentation**
Left heart		
Left atrial end-diastolic volume, mL	86 (65–126)	90 (69–124)
Left atrial end-systolic volume, mL	36 (24–63)	37 (27–63)
Left atrial stroke volume, mL	46 (35–59)	51 (39–64)
Left atrial ejection fraction, %	56 (46–64)	58 (48–65)
Left atrial global longitudinal strain, %	-15 (-21 to -9)	-21 (-27 to -15)
Left ventricular end-diastolic volume, mL	155 (134–192)	156 (136–200)
Left ventricular end-systolic volume, mL	66 (48–92)	63 (49–95)
Left ventricular stroke volume, mL	86 (72–104)	91 (75–109)
Left ventricular mass, g	133 (109–167)	133 (108–167)
Left ventricular ejection fraction, %	56 (47–64)	58 (50–66)
Left ventricular peak ejection rate, mL/s	417 (367–493)	446 (401–553)
Left ventricular peak filling rate, mL/s	448 (331–549)	487 (370–610)
Left ventricular cardiac output, mL/min	5,506 (4,888–6,910)	5,878 (5,046–7,178)
Left ventricular global longitudinal strain, %	-17 (-20 to -13)	-20 (-22 to -15)
Right heart		
Right atrial end-diastolic volume, mL	72 (52–87)	72 (54–88)
Right atrial end-systolic volume, mL	36 (25–48)	33 (26–44)
Right atrial stroke volume, mL	31 (23–40)	33 (26–45)
Right atrial ejection fraction, %	48 (40–55)	51 (42–57)
Right atrial global longitudinal strain, %	-16 (-21 to -11)	-22 (-26 to -18)
Right ventricular end-diastolic volume, mL	76 (57–100)	84 (61–106)
Right ventricular end-systolic volume, mL	24 (16–35)	28 (20–38)
Right ventricular stroke volume, mL	50 (38–63)	54 (41–68)
Right ventricular ejection fraction, %	68 (61–73)	66 (60–71)
Right ventricular cardiac output, mL/min	3,331 (2,505–4,158)	3,608 (2,671–4,336)
Right ventricular global longitudinal strain, %	-30 (-34 to -23)	-29 (-34 to -25)

Data are given as median (interquartile range)

### Correlation between AI and manual methods

**Fig. 2:**
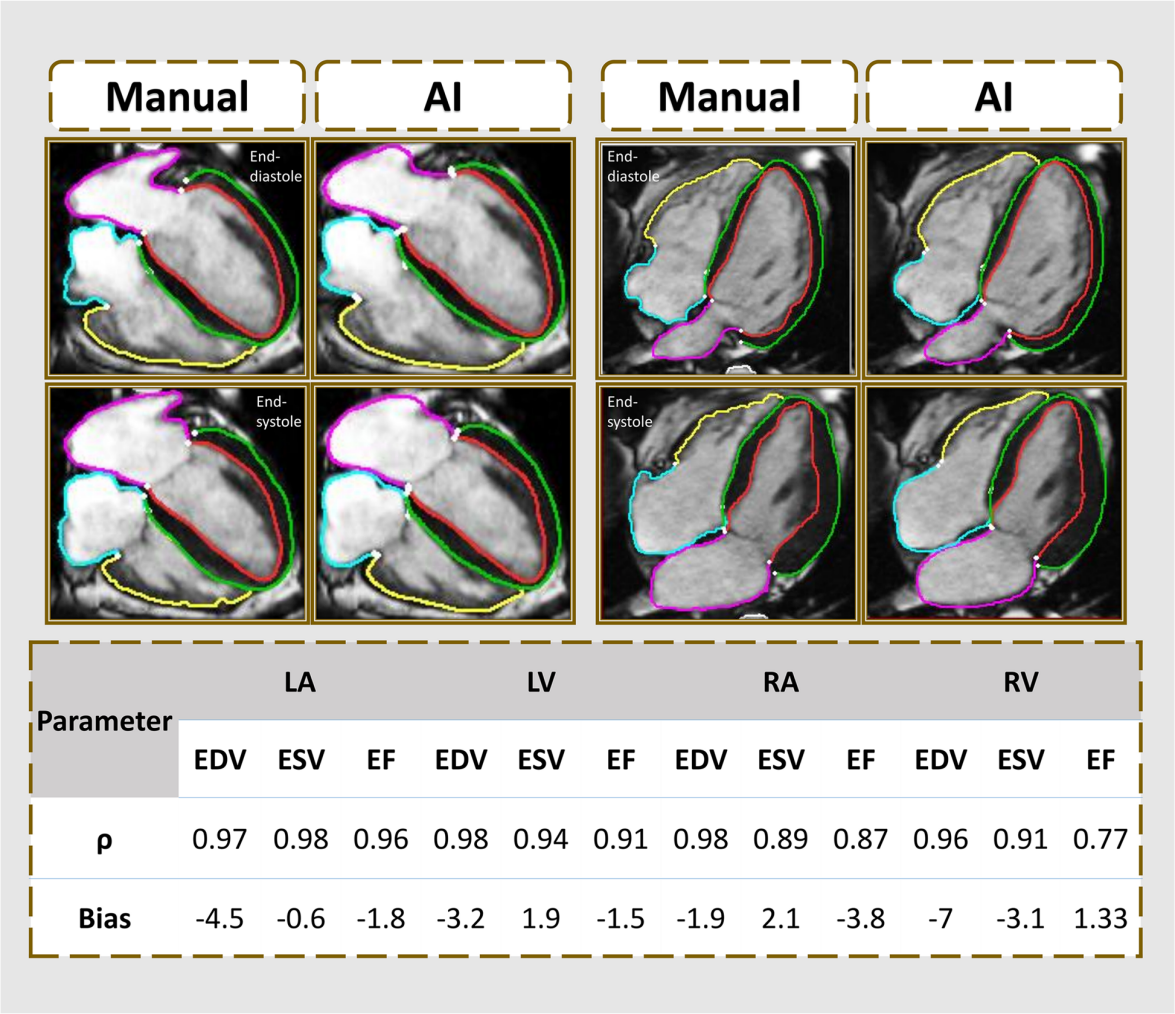
Examples of the manual and automated four-chamber segmentation methods and their correlation. The AI method segments all time frames; however, only end-diastole and end-systole frames are demonstrated. The coloured contours are as follows: green for the LV epicardium, red for the LV endocardium, pink for the LA, yellow for the RV endocardium, and turquoise contours for the RA. *AI* Artificial intelligence, *EDV* End-diastolic volume, *EF* Ejection fraction, *ESV* End-systolic volume, *LA* Left atrium, *LV* Left ventricle, *ρ* Spearman correlation coefficient, *RA* Right atrium, *RV* Right ventricle

The results regarding the correlation, CoV, and Bland–Altman analyses are provided in Fig. 2, Table 3 and Supplemental Fig. S1 and S2. Notably, left heart parameters, including LA EDV, LA end-systolic volume (ESV), LA SV, LA EF, LV EDV, LV ESV, LV SV, and LV EF, demonstrated strong correlations (LA EDV, *ρ* = 0.97; LA ESV, *ρ* = 0.98; LA SV, *ρ* = 0.94; LA EF, *ρ* = 0.96; LV EDV, *ρ* = 0.98; LV ESV, *ρ* = 0.94; LV SV, *ρ* = 0.94; LVEF, *ρ* = 0.91), with small bias (LA EDV -4.5 mL; LA ESV -0.6 mL; LA SV -4.3 mL; LA EF -1.8%; LV EDV -3.2 mL; LV ESV 1.9 mL; LV SV -5.1 mL; LVEF -1.5%) signifying good agreement between measurement techniques. The highest within-subject CoVs for the left heart were observed for LA SV (13%) and LV peak filling rate (27%), suggesting greater variability in measurements within individual subjects. Moreover, right heart parameters, including RA EDV, RA SV, RV EDV, RV ESV and RV SV, exhibited strong correlations (*ρ* = 0.98, *ρ* = 0.91, *ρ* = 0.96, *ρ* = 0.91, and *ρ* = 0.92, respectively) and minimal bias (-2 mL, -3.9 mL, -7 mL, -3.1 mL, and -3.9 mL, respectively). The highest within-subject CoVs for the right heart were observed for RA SV (21%) and RV ESV (29%), indicating high variability in measurements within individual subjects. In contrast, strain analysis for RA and RV global longitudinal strain (GLS) showed weak to moderate correlations (*ρ* = 0.66 and *ρ* = 0.58, respectively) and wider limits of agreement. The results remained the same after excluding the two failed cases from the quality control assessment.

**Table 3:** Correlation and coefficient of variation between AI-generated and manual segmentations for all four chamber parameters

Variable	Correlation (*ρ*)	*p*-value	CoV %
Left heart			
Left atrial end-diastolic volume, mL	0.97	< 0.001	6.4
Left atrial end-systolic volume, mL	0.98	< 0.001	7.2
Left atrial stroke volume, mL	0.94	< 0.001	13.1
Left atrial ejection fraction, %	0.96	< 0.001	6.9
Left atrial global longitudinal strain, %	0.74	< 0.001	-39.6
Left ventricular end-diastolic volume, mL	0.98	< 0.001	4.0
Left ventricular end-systolic volume, mL	0.94	< 0.001	11.2
Left ventricular stroke volume, mL	0.94	< 0.001	9.6
Left ventricular mass, g	0.93	< 0.001	7.3
Left ventricular ejection fraction, %	0.91	< 0.001	6.9
Left ventricular peak ejection rate, mL/s	0.84	< 0.001	13.0
Left ventricular peak filling rate, mL/s	0.86	< 0.001	26.7
Left ventricular cardiac output, mL/min	0.93	< 0.001	11.1
Left ventricular global longitudinal strain, %	0.75	< 0.001	-14.8
Right heart			
Right atrial end-diastolic volume, mL	0.98	< 0.001	8.2
Right atrial end-systolic volume, mL	0.89	< 0.001	12.8
Right atrial stroke volume, mL	0.91	< 0.001	20.5
Right atrial ejection fraction, %	0.87	< 0.001	14.6
Right atrial global longitudinal strain, %	0.66	< 0.001	-42.4
Right ventricular end-diastolic volume, mL	0.96	< 0.001	12.0
Right ventricular end-systolic volume, mL	0.91	< 0.001	29.4
Right ventricular stroke volume, mL	0.92	< 0.001	11.5
Right ventricular ejection fraction, %	0.77	< 0.001	8.6
Right ventricular cardiac output, mL/min	0.93	< 0.001	11.8
Right ventricular global longitudinal strain, %	0.58	< 0.001	-19.5

*CoV* Coefficient of variation (within-subject standard deviation method), *ρ* Spearman rank correlation coefficient

### Repeatability

All AI-generated four-chamber volumes showed excellent repeatability without bias on Pearson correlation analysis (*r* = 0.99−1.00). Only LV peak ejection rate and peak filling rate showed minimum bias (-1.5 mL/s and -1 mL/s, respectively). Within-subject CoVs were < 1% in all volumetric analyses apart from LV peak ejection rate (2%) and LV peak filling rate (1%), showing overall excellent consistency in volumetric analyses. Full results of the automated four-chamber repeatability analysis are shown in Supplemental Table S2.

### Agreement with short-axis volumetric measurements

Left ventricular EDV and ESV exhibited significant strong positive correlations and a bias towards lower values in the four-chamber compared to short-axis analysis (LV ESV, *r* = 0.86, bias =  -12.5 mL, *p* = 0.001; LV ESV, *r* = 0.91, bias =  -14.8 mL, *p* < 0.001). LV mass and LV EF showed a strong negative correlation (*r* = 0.89 and *r* = 0.88) with a bias towards higher values in the short-axis than four-chamber measurements (bias = 6.9 g and 4.6%, respectively). However, LV peak ejection rate and peak filling rate demonstrated a moderate positive correlation with the short-axis measurements (*r* = 0.52 for both) and bias of -37.4 mL/s and 43.7 mL/s, respectively. Additionally, RV measurements consistently indicated larger mean volumes (RV EDV 155 mL *versus* 86 mL; RV ESV 69 mL *versus* 31 mL; RV SV 87 mL *versus* 55 mL) and lower ejection fraction (57% *versus* 65%) in the short-axis compared to the four-chamber analyses.

**Fig. 3:**
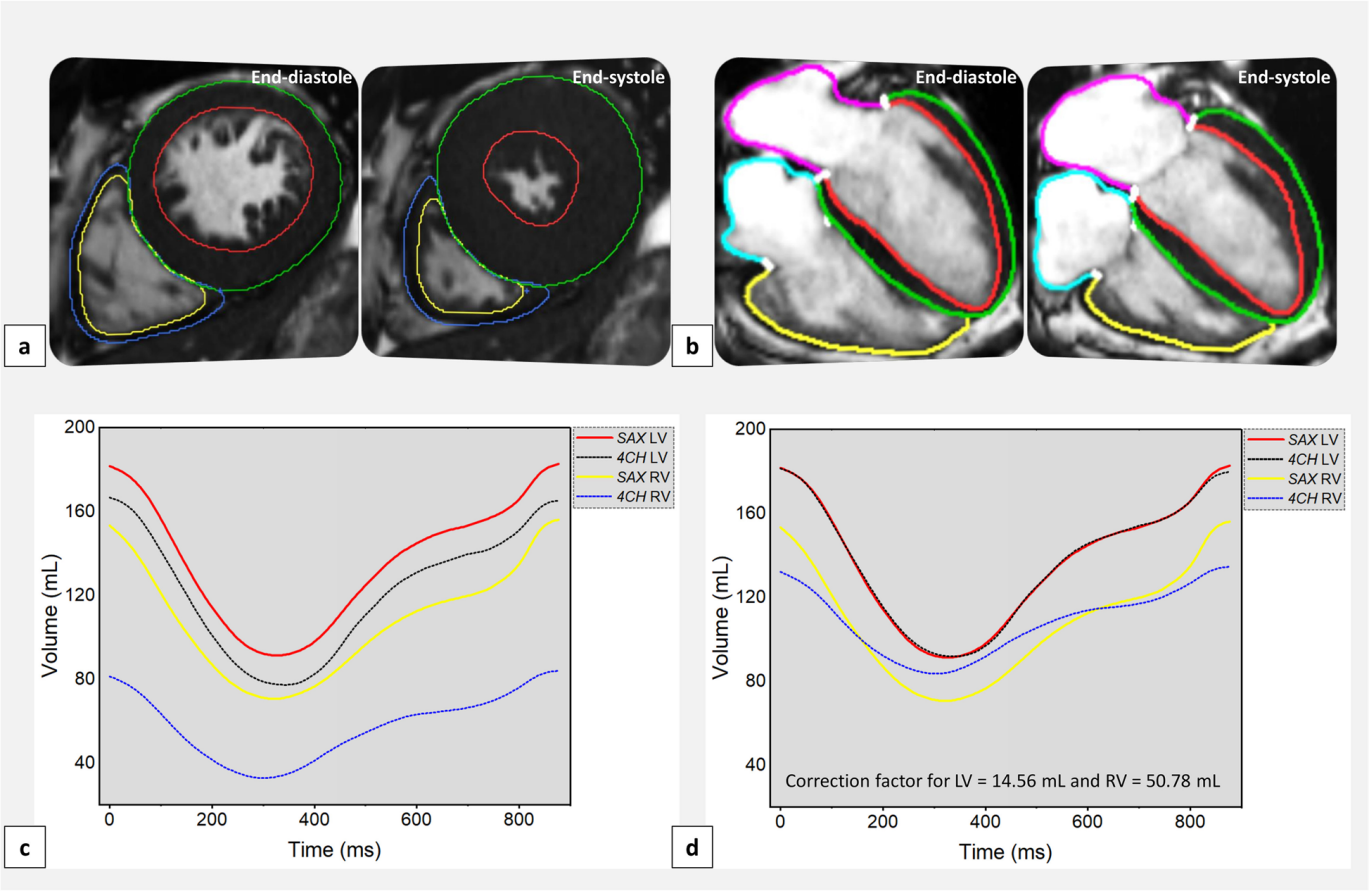
Quantification results of mean LV and RV volumes of automated four-chamber and short-axis segmentation methods over time. **a **AI-generated contours of short-axis cine stack of images using standard endocardial and epicardial contour methods. **b **AI-generated four-chamber cine segmentation contours using standard endocardial and epicardial contours methods. **c** The AI-generated four-chamber cine segmentation results yielded slightly lower left and right ventricular volumes than the ground truth. **d** Quantification results of automated four-chamber mean left and right ventricular volumes after applying 14.56 mL and 50.78 mL correction factors and short-axis segmentation results over time. The AI-generated four-chamber cine LV segmentation results were similar, and RV yielded slightly higher volumes than the ground truth. *AI* Artificial intelligence, *4CH* Four-chamber, *LV* Left ventricle, *RV* Right ventricle, SAX Short-axis

Two correction factors for LV (14.56 mL) and RV (50.78 mL) four-chamber volumetric analysis were proposed. After applying the correction factors, there were no significant differences and minimal bias between automated four-chamber and short-axis measurements for LV EDV (bias = 2.04 mL, *p* = 0.493) and LV ESV (bias = -0.26 mL, *p* = 0.903). However, the results remained similar for the RV measurements, with bias reducing from -69 mL to -18.7 mL for RV EDV and from -37.6 mL to 13.2 mL for RV ESV. Mean LV and RV volumetric curves, correlation, and Bland–Altman results before and after applying the correction factor of AI-generated four-chamber and short-axis segmentations are shown in Fig. 3, Supplemental Tables S3 and S4, and Supplemental Fig. S3 and S4.

### Quality control assessment

In our validation cohort, 88 of 101 cases were satisfactory, and 11 (11%) had suboptimal contours. Seven of the 11 misplaced contours were in the RA, mainly because of the off-plane four-chamber slice disposition. Four suboptimal cases were in the LA and LV regions. The LV suboptimal contours were due to including the LV outflow tract as part of the LV volume. Two of the 101 cases failed due to severe image artefacts and, in one case, due to the inclusion of pericardial fat in the RV contours (Supplemental Table S5).

### Survival analysis

Patients who died had greater mean LV mass in the manual and AI assessments (both 164 g *versus* 137 g, *p* = 0.041). Moreover, mean LA EF was significantly lower in patients who died during follow-up in manually-derived (44% *versus* 56%, *p* = 0.004) and AI-derived (45% *versus* 58%, *p* = 0.002) segmentations. Additionally, in the AI-generated segmentation analysis, mean LA SV (42 mL *versus* 55 mL) and RA EF (42 mL *versus* 52 mL) were higher, and LA GLS (-16% *versus* -23%) and RA GLS (-17% versus -24%) were lower in patients who died (all *p* < 0.05). The AI-generated segmentation analysis and the manually generated segmentation results are demonstrated in Supplemental Tables S6 and S7.

At univariable Cox regression analysis, only LV mass and LA EF were significantly associated with death in manual segmentation (LV mass, hazard ratio (HR) = 1.01, *p* = 0.047; LA EF, HR = 0.96, *p* = 0.004). On AI-derived segmentation, four left heart (LV mass, LA SV, LA GLS, and LA EF) and two right heart (RA GLS and RA EF) CMR parameters were associated with death (LV mass, HR = 1.01, *p* = 0.046; LA SV, HR = 0.96, *p* = 0.017; LA GLS, HR = 1.07, *p* = 0.017; LA EF, HR = 0.96, *p* = 0.002; RA GLS, HR = 1.07, *p* = 0.011; RA EF, HR = 0.96, *p* = 0.021). On stepwise multivariable Cox-proportional-hazard analysis, only LA EF demonstrated an independent association with death in both manual (*β* =  -0.04, standard error = 0.01, HR = 0.96, *p* = 0.003) and AI analyses (*β* =  -0.05, standard error = 0.01, HR = 0.96, *p* < 0.001) (Supplemental Tables S8 and S9).

**Fig. 4:**
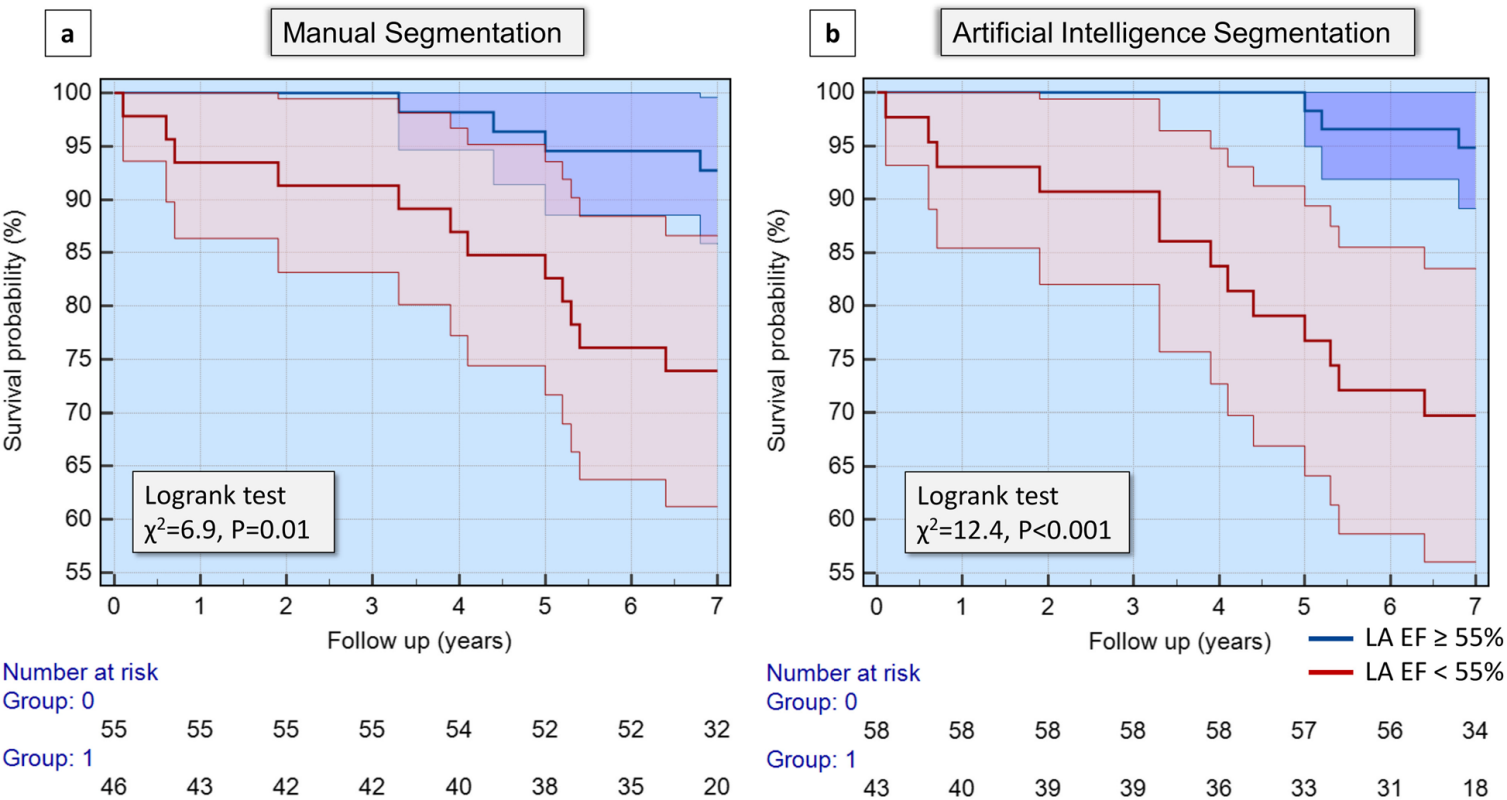
Survival analysis. **a** Kaplan–Meier analysis demonstrates that patients with left atrial ejection fraction < 55% had a higher risk of death. **b **The risk of death is higher when using artificial intelligence-generated segmentation. *EF* Ejection fraction, *LA* Left atrium

**Fig. 5:**
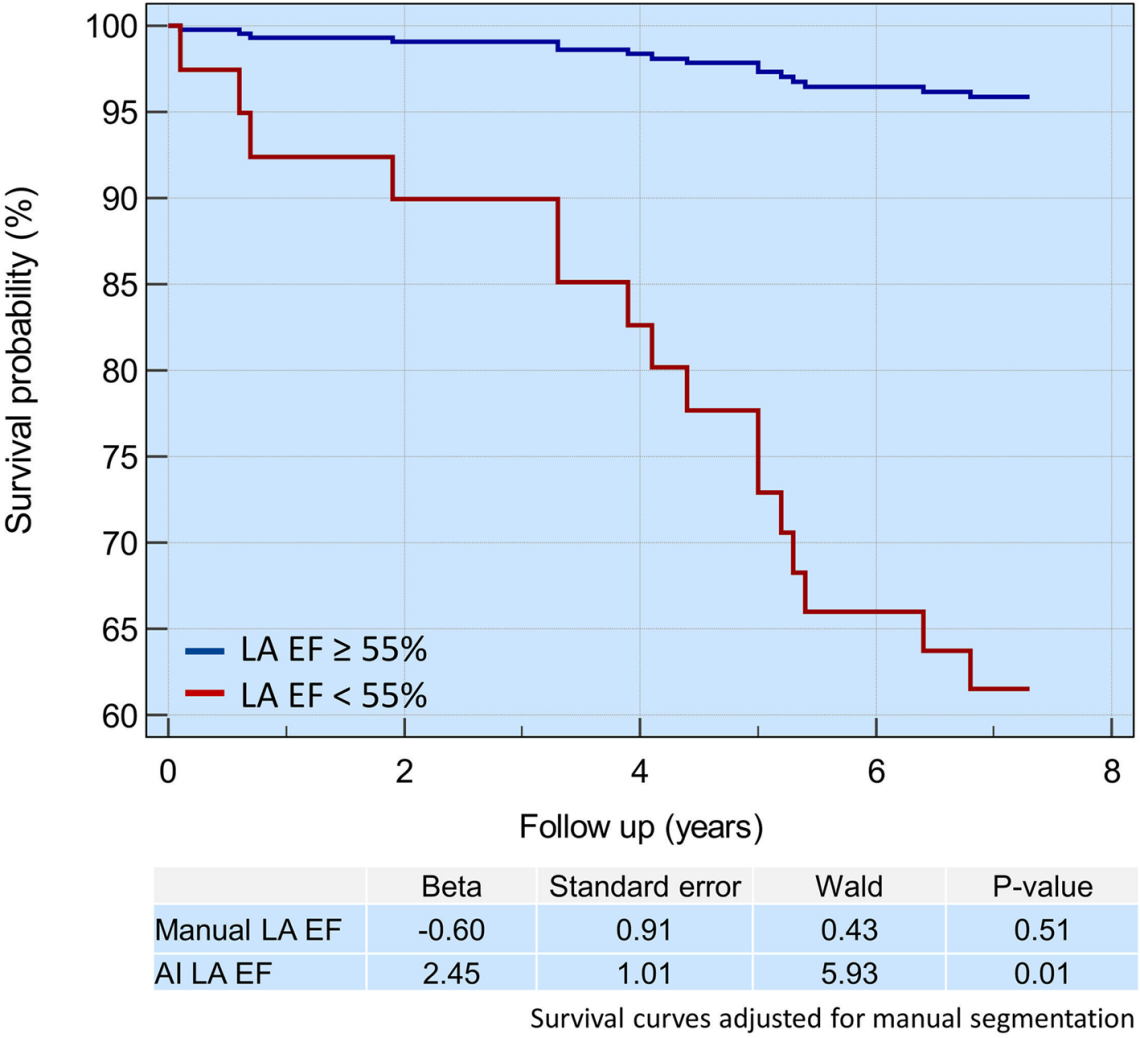
Kaplan-Meir analysis demonstrates that AI-generated LA ejection fraction < 55% is independently associated with risk of death after adjusting for manual LA EF in the regression model. *AI* Artificial intelligence, *EF* Ejection fraction, *LA* Left atrium

At Kaplan–Meier analysis, patients with LA EF < 55% demonstrated a significantly higher association with death, with the risk of death being more significant in AI-generated CMR segmentation (95%, *χ*^2^ = 12.4, *p* < 0.001) than in manual segmentation (92%, *χ*^2^ = 6.9, *p* = 0.009) (Fig. 4). Moreover, after adjusting for manual LA EF in the regression model, AI-generated LA EF < 55% was independently associated with the risk of death (Fig. 5).

## Discussion

In this study, we developed a time-resolved, AI deep-learning-based segmentation solution for all four chambers of the heart using multicentre and multivendor four-chamber cine data. In an external validation cohort of 101 CMR scans, we observed good agreement and excellent repeatability for left and right heart volumetric assessments. However, longitudinal strain parameters only demonstrated moderate agreement. Additionally, we found that the AI four-chamber cine analysis significantly underestimates LV and LV volume systemically compared with the ground truth based on analysis of a short-axis cine stack. In this study, we provide correction factors for both LV and RV volumes using automated four-chamber analysis. In this heterogenous real-world all-comers CMR data, we noted AI segmentation informed LA EF was independently associated with all-cause mortality.

Over the last few years, many studies have used different research and commercial software solutions to develop and validate automated segmentation models for four-chamber cine CMR sequences [3, 12, 13, 23, 24]. While showing potential, these studies had several limitations. Bai et al. [3] trained an AI-segmentation model using data from 3,782 healthy subjects, tested it on 600 (541 healthy and 39 cardiovascular disease patients), compared automated LA and RA four-chamber segmentation with manually acquired data and demonstrated that the accuracy of AI-segmentation measures is in agreement with human expert performance (mean Dice similarity coefficient of 0.95 and 0.96 for LA and RA, respectively). However, they did not report any volumetric results, and their focus was only on the atria. Ruijsink et al. [23] developed a fully automated four-chamber cine functional CMR assessment model using 3,975 single-centre and single-vendor data from healthy volunteers and patients with various cardiovascular diseases to train a model. They compared the results of LV strain analysis obtained from their AI model with those of manual analysis on a validation cohort of 100 healthy controls and cardiomyopathy patients [23]. Similar to our LV strain analysis results (*r* = 0.82, bias = 1.89%, *p* < 0.001), they achieved a good correlation between manual and automated LV strain analysis (*r* = 0.89, bias = 1.03%, *p* < 0.001) [23]. The results of both studies were produced using the commercially available software cvi42 [3, 23]. However, both models used the UK biobank, single-vendor (1.5-T Siemens scanner), and homogeneous CMR dataset to train the algorithms, limiting their broader applicability.

Moreover, a study by Shahzad et al. [12] investigated the feasibility, accuracy, and agreement of an AI-segmentation pipeline for LV volumetric parameters with both manual annotations and short-axis cine. Using the same research software (MASS) as in the current study, they trained and tested an AI model combining the four-chamber and two-chamber cine scans using multicentre, single-vendor (3-T Philips scanner) data of 145 healthy controls and subjects with various cardiac conditions [12]. Similar to our study, they observed a very good agreement between AI and manual segmentation results of left ventricular EF, SV, EDV and ESV (*r* = 0.84−0.97) [12]. Furthermore, when comparing AI-segmented four-chamber to short-axis volumetric measurements, a strong positive correlation was also seen for the same LV parameters (*r* = 0.87−0.99) [12]. Our study achieved similar results in an external validation cohort, showing that automated four-chamber segmentation agrees with the ground truth. The bias reduced substantially after applying the correction factor for LV EDV (2.04 mL), LV ESV (-0.26 mL), RV EDV (-18.7 mL), and RV ESV (13.2 mL), further supporting the claim that functional LV parameters obtained from the four-chamber analysis are as accurate as the short-axis segmentation [12, 25]. Even as four-chamber analysis relies on geometric assumptions, the benefit of viewing the basal and apical margins of the left ventricle perpendicularly may compensate for the drawback of using calculated data to replace real measurements partially [26].

Gonzales et al. [13] developed and tested a time-resolved, four-chamber cine, fully automated left atrial segmentation model on 37 patients with various cardiovascular diseases from a single centre and single vendor (1.5-T Siemens) scanner. Using the medical image analysis software “Segment”, they achieved good agreement between AI and manually-segmented LA volumetric analysis, with *r* = 0.98, 0.97, 0.96, and 0.92 for EDV, ESV, EF and GLS, respectively [13]. The only difference between our results and theirs is our relatively low correlation between automated and manual segmentation results for LA GLS (*ρ* = 0.74), as our AI model tends to overestimate strain measures. However, there was no mention of the number of cases used for model training by these authors. Additionally, the testing dataset had a relatively small sample size (*n* = 37) [13]. One recent study used 3,925 subjects from the UK biobank for training and 600 subjects for testing an automated three-dimensional four-chamber CMR image analysis model of all heart chambers [24]. They observed a strong agreement between the automated and manual methods for LV and RV measurements (*r* = 0.87−0.93) and a slightly lower agreement in LA (*r* = 0.76 and 0.81) and RA (*r* = 0.76 and 0.86) EDV and ESV, respectively [24]. Our AI model was trained using a multicentre multivendor cohort and tested on an external dataset, achieving a stronger correlation with manual analysis for LA EDV (*ρ* = 0.97) and ESV (*ρ* = 0.98) and RA EDV (*ρ* = 0.98) and ESV (*ρ* = 0.89). Although their three-dimensional model was trained using a single centre (UK biobank) and vendor (1.5-T Siemens scanner) dataset, the testing cohort was substantially larger (*n* = 600) than that of our study [24].

The four-chamber view is advantageous in scenarios requiring a comprehensive assessment of both ventricles and atria. A well-executed four-chamber cine is of significant clinical value as it facilitates a comparative analysis of the LA and other chambers. On the other hand, segmental wall motion and left ventricular mass are usually assessed using the short-axis view. It might, however, be restricted in some circumstances, such as difficulty in evaluating left ventricular segmental wall motion using established guidelines and evaluation of ventricular mass due to obscure epicardial and endocardial borders in the apical slices of the heart [27]. Moreover, the four-chamber view enables the assessment of the pericardium, subcutaneous adipose tissue, and descending aorta. Therefore, a four-chamber analysis can be invaluable in situations where high-quality cines in other views are unavailable due to arrhythmias or other issues.

This study enhances the clinical applicability of four-chamber analysis by introducing correction factors for both LV and RV volumetric assessments to compensate for the underestimation compared with the reference standard short-axis stack measurements. There are several clinical scenarios where a four-chamber cine could potentially be more beneficial than a short-axis stack assessment. One of the primary advantages of a four-chamber cine is that it requires only a single breath-hold and a few seconds to acquire. This feature could be particularly valuable for patients who are claustrophobic or have poor echocardiographic views. Typically, a four-chamber cine is one of the first cine sequences to be acquired in CMR protocols. Therefore, even if a patient is claustrophobic, evaluating the function of all four chambers is still possible. Another advantage of a four-chamber cine is that it allows for a longitudinal functional assessment, which is not possible with a short-axis stack. Furthermore, spatial misalignment between the slices in short-axis cines can lead to errors in functional and volumetric calculations. In such cases, a four-chamber cine could potentially guide management decisions. Lastly, a four-chamber cine volumetric and functional assessment can serve as an internal validation check on a short-axis cine assessment. In instances where there is a substantial disagreement, the reporter has the option to revisit the contours, whether they were done manually or using AI deep learning contours.

This study has limitations. First, the four-chamber analysis depends on acquisition. If wrongly planned, it can lead to underestimating or overestimating volumes. However, we had clear acquisition protocols, which likely reduced this error. Second, although our model was trained on a large multicentre multivendor heterogeneous data, our external validation single centre and vendor cohort did not include complex variations of data in clinical practice, such as congenital heart disease, and was relatively small in size. Future studies should test the model on larger, multicentre, multivendor, and other disease types commonly seen in clinical practice to determine wider applicability. Finally, our quality control was performed manually by an expert observer. Future studies should look at automating this process.

In conclusion, fully automated four-chamber CMR is feasible, reproducible and appears to have the same prognostic value as manual analysis in real-world CMR. Four-chamber analysis systemically underestimates LV and RV volumes. After applying the correction factors, LV volumes by four-chamber segmentation are comparable to short-axis volumetric assessment.

### Supplementary Information

Supplementary Material 1: Table S1. Normal distribution test for CMR variables with the reference manual segmentation. Shapiro–Wilk test (a significant *p*-value demonstrates not normally distributed data). Table S2. AI repeatability results of fully automated four-chamber CMR analysis. Table S3. CMR characteristics, correlation and Bland-Altman results of AI-generated four-chamber and short-axis segmentations. Table S4. CMR characteristics, correlation and Bland-Altman results of AI-generated four-chamber and short-axis segmentations after applying the correction factor (LV = 14.5623 mL; RV = 50.7676 mL). Table S5. Quality control assessment of the external validation cohort (*n* = 101). Table S6. Left and right heart CMR functional assessment by manual analysis. Table S7. Left and right heart CMR functional assessment by automated analysis. Table S8. Univariable and stepwise multivariable Cox regression analysis of all four chambers by manual examination. Table S9. Univariable and stepwise multivariable Cox regression analysis of all four chambers by automated analysis. Fig. S1. Bland-Altman plots demonstrating the degree of agreement between manual segmentation and AI four-chamber analysis of the left atrium and left ventricle. (a) LA EDV left atrial enddiastolic volume. (b) LA ESV left atrial end-systolic volume. (c) LA SV left atrial systolic volume. (d) LA EF left atrial ejection fraction. (e) LV EDV left ventricular end-diastolic volume. (f) LV ESV left ventricular end-systolic volume. (g) LV SV left ventricular systolic volume. (h) LV EF left ventricular ejection fraction. Fig. S2. Bland-Altman plots demonstrating the degree of agreement between manual segmentation and AI four-chamber analysis of the right atrium (a, b, c, d) and right ventricle (e, f, g, h). (a) RA EDV right atrial end-diastolic volume. (b) RA ESV right atrial end-systolic volume. (c) RA SV right atrial systolic volume. (d) RA EF right atrial ejection fraction. (e) RV EDV right ventricular end-diastolic volume. (f) RV ESV right ventricular end-systolic volume. (g) RV SV right ventricular systolic volume. (h) RV EF right ventricular ejection fraction. Fig. S3. Bland-Altman plots demonstrating the degree of agreement between AI-generated fourchamber and short-axis segmentations. (a) LV EDV left ventricular end-diastolic volume. (b) LV ESV left ventricular end-systolic volume. (c) LV SV left ventricular systolic volume. (d) LV EF left ventricular ejection fraction. (e) RV EDV right ventricular end-diastolic volume. (f) RV ESV right ventricular endsystolic volume. (g) RV SV right ventricular stroke volume. (h) RV EF right ventricular ejection fraction. Fig. S4. Bland-Altman plots demonstrating the degree of agreement between AI-generated fourchamber and short-axis segmentations after applying the correction factor (LV = 14.5623 mL; RV = 50.7676 mL). (a) LV EDV left ventricular end-diastolic volume. (b) LV ESV left ventricular end-systolic volume. (c) RV EDV right ventricular end-diastolic volume. (d) RV ESV right ventricular end-systolic volume

Supplementary Material 2. Examples of quality control assessment results of the validation cohort: (a) Satisfactory, (b) suboptimal, and (c) failure categorisations.

## Data Availability

The datasets generated and analysed during the current study are not publicly available. Access to the raw images of patients is not permitted since specialised postprocessing imaging-based solutions can identify the study patients in the future. Data are available from the corresponding author upon reasonable request.

## Abbreviations

AI: Artificial intelligence
bSSFP: Balanced steady-state free precession
CMR: Cardiac magnetic resonance imaging
CoV: Coefficient of variation
EDV: End-diastolic volume
EF: Ejection fraction
ESV: End-systolic volume
GLS: Global longitudinal strain
HR: Hazard ratio
LA: Left atrium
LV: Left ventricle
RA: Right atrium
RV: Right ventricle
SD: Standard deviation
SV: Stroke volume
