# Supplementary material for: Development and validation of AI-derived segmentation of four-chamber cine cardiac magnetic resonance
Source: Eur Radiol Exp. 2024 Jul 12;8:77. doi: 10.1186/s41747-024-00477-7 (PMC11239622; doi:10.1186/s41747-024-00477-7)
Supplement: Supplementary file 1 — Supplementary Material 1: Table S1. Normal distribution test for CMR variables with the reference manual segmentation. Shapiro–Wilk test (a significant p-value demonstrates not normally distributed data). Table S2. AI repeatability results of fully automated four-chamber CMR analysis. Table S3. CMR characteristics, correlation and Bland-Altman results of AI-generated four-chamber and short-axis segmentations. Table S4. CMR characteristics, correlation and Bland-Altman results of AI-generated four-chamber and short-axis segmentations after applying the correction factor (LV = 14.5623 mL; RV = 50.7676 mL). Table S5. Quality control assessment of the external validation cohort (n = 101). Table S6. Left and right heart CMR functional assessment by manual analysis. Table S7. Left and right heart CMR functional assessment by automated analysis. Table S8. Univariable and stepwise multivariable Cox regression analysis of all four chambers by manual examination. Table S9. Univariable and stepwise multivariable Cox regression analysis of all four chambers by automated analysis. Fig. S1. Bland-Altman plots demonstrating the degree of agreement between manual segmentation and AI four-chamber analysis of the left atrium and left ventricle. (a) LA EDV left atrial enddiastolic volume. (b) LA ESV left atrial end-systolic volume. (c) LA SV left atrial systolic volume. (d) LA EF left atrial ejection fraction. (e) LV EDV left ventricular end-diastolic volume. (f) LV ESV left ventricular end-systolic volume. (g) LV SV left ventricular systolic volume. (h) LV EF left ventricular ejection fraction. Fig. S2. Bland-Altman plots demonstrating the degree of agreement between manual segmentation and AI four-chamber analysis of the right atrium (a, b, c, d) and right ventricle (e, f, g, h). (a) RA EDV right atrial end-diastolic volume. (b) RA ESV right atrial end-systolic volume. (c) RA SV right atrial systolic volume. (d) RA EF right atrial ejection fraction. (e) RV EDV right ventricular end- [file 41747_2024_477_MOESM1_ESM.pdf]

Development and validation of AI-derived segmentation of four-chamber cine cardiac magnetic resonance

ELECTRONIC SUPPLEMENTARY MATERIAL

Table S1. Normal distribution test for CMR variables with the reference manual segmentation. Shapiro–Wilk test (a significant P-value demonstrates not normally distributed data).

Variable	***p-value***
Left atrial end-diastolic volume, mL	< 0.001
Left atrial end-systolic volume, mL	< 0.001
Left atrial stroke volume, mL	0.012
Left atrial ejection fraction, %	< 0.001
Left atrial global longitudinal strain, %	0.005
Left ventricular end-diastolic volume, mL	< 0.001
Left ventricular end-systolic volume, mL	< 0.001
Left ventricular stroke volume, mL	0.021
Left ventricular mass, g	0.003
Left ventricular ejection fraction, %	0.036
Left ventricular peak ejection rate, mL/sec	< 0.001
Left ventricular peak filling rate, mL/sec	< 0.001
Left ventricular cardiac output, mL/min	< 0.001
Left ventricular global longitudinal strain, %	0.566
Right atrial end-diastolic volume, mL	< 0.001
Right atrial end-systolic volume, mL	< 0.001
Right atrial stroke volume, mL	0.029
Right atrial ejection fraction, %	0.002
Right atrial global longitudinal strain, %	0.507
Right ventricular end-diastolic volume, mL	0.005
Right ventricular end-systolic volume, mL	< 0.001
Right ventricular stroke volume, mL	0.010
Right ventricular ejection fraction, %	0.001
Right ventricular cardiac output, mL/min	0.016

Right ventricular global longitudinal strain, %	0.115

Table S2. AI repeatability results of fully automated four-chamber CMR analysis.

Variable	*Correlation (ρ)*	*CoV %*	*Bias*	*p-value*
**Left heart**				
Left atrial end-diastolic volume, mL	1	0	0	< 0.001
Left atrial end-systolic volume, mL	1	0	0	< 0.001
Left atrial stroke volume, mL	1	0	0	< 0.001
Left atrial ejection fraction, %	1	0	0	< 0.001
Left ventricular end-diastolic volume, mL	1	0.2	0.2	< 0.001
Left ventricular end-systolic volume, mL	1	0.8	0.9	< 0.001
Left ventricular stroke volume, mL	1	0.3	0.4	< 0.001
Left ventricular mass, g	1	0	0	< 0.001
Left ventricular ejection fraction, %	1	0.5	0.5	< 0.001
Left ventricular peak ejection rate, mL/sec	0.992	2.1	2.2	< 0.001
Left ventricular peak filling rate, mL/se**c**	0.996	1.4	1.4	< 0.001
Left ventricular cardiac output, mL/min	1	0.4	0.4	< 0.001
**Right heart**				
Right atrial end-diastolic volume, mL	1	0	0	< 0.001
Right atrial end-systolic volume, mL	1	0	0	< 0.001
Right atrial stroke volume, mL	1	0	0	< 0.001
Right atrial ejection fraction, %	1	0	0	< 0.001
Right ventricular end-diastolic volume, mL	1	0	0	< 0.001
Right ventricular end-systolic volume, mL	1	0	0	< 0.001
Right ventricular stroke volume, mL	1	0	0	< 0.001
Right ventricular ejection fraction, %	1	0	0	< 0.001
Right ventricular cardiac output, mL/min	1	0	0	< 0.001

CoV Coefficient of variation (within-subject standard deviation method), ρ Spearman rank correlation coefficient.

Table S3. CMR characteristics, correlation and Bland-Altman results of AI-generated four-chamber and short-axis segmentations.

Variable	***Four-chamber***	***SAX***	***R***	***Bias***	***p*-value**
**Left Heart**					
Left ventricular end-diastolic volume, mL	172 ± 54.3	185.3 ± 56.8	0.86	-12.5	< 0.001
Left ventricular end-systolic volume, mL	77.5 ± 44.7	92.8 ± 51.1	0.91	-14.8	< 0.001
Left ventricular stroke volume, mL	94.7 ± 27.4	92.5 ± 25.8	0.73	2.3	0.241
Left ventricular mass, g	141.2 ± 48.9	133.3 ± 36.9	0.89	6.9	0.002
Left ventricular ejection fraction, %	57 ± 12.6	52.2 ± 12.9	0.88	4.6	< 0.001
Left ventricular peak ejection rate, mL/sec	482 ± 146	518 ± 136	0.52	-37.4	0.009
Left ventricular peak filling rate, mL/sec	513 ± 241	468 ± 150	0.52	43.7	0.041
Left ventricular cardiac output, mL/min	6,346 ± 1,981	6,053 ± 1,663	0.68	278	0.070
**Right Heart**					
Right ventricular end-diastolic volume, mL	85.8 ± 31.3	155.2 ± 41.8	0.70	-69	< 0.001
Right ventricular end-systolic volume, mL	30.9 ± 16.1	68.6 ± 28.5	0.79	-37.6	< 0.001
Right ventricular stroke volume, mL	54.8 ± 19.8	86.6 ± 25	0.58	-31.8	< 0.001
Right ventricular ejection fraction, %	65 ± 9.8	56.7 ± 11	0.65	8.2	< 0.001
Right ventricular cardiac output, mL/min	3,668 ± 1,352	5,636 ± 1,519	0.52	-1984	< 0.001

Data are given as mean ± standard deviation.

Table S4. CMR characteristics, correlation and Bland-Altman results of AI-generated four-chamber and short-axis segmentations after applying the correction factor (LV=14.5623 mL; RV=50.7676 mL).

Variable	***Four-chamber***	***SAX***	***R***	***Bias***	***p*-value**
**Left heart**					
Left ventricular end-diastolic volume, mL	186.8 ± 54.3	185.3 ± 56.8	0.86	2.04	0.493
Left ventricular end-systolic volume, mL	92.1 ± 44.7	92.8 ± 51.1	0.91	-0.26	0.903
Left ventricular stroke volume, mL	94.7 ± 27.4	92.5 ± 25.8	0.73	2.3	0.241
Left ventricular cardiac output, mL/min	6,346 ± 1,981	6,053 ± 1,663	0.68	278	0.070
**Right heart**					
Right ventricular end-diastolic volume, mL	136.6 ± 31.3	155.2 ± 41.8	0.70	-18.7	< 0.001
Right ventricular end-systolic volume, mL	81.7 ± 16.1	68.6 ± 28.5	0.79	13.2	< 0.001
Right ventricular stroke volume, mL	54.8 ± 19.8	86.6 ± 25	0.58	-31.8	< 0.001
Right ventricular cardiac output, mL/min	3,668 ± 1,352	5,636 ± 1,519	0.52	-1984	< 0.001

Data are given as mean ± standard deviation.

Table S5. Quality control assessment of the external validation cohort (*n* = 101).

**AI-derived segmentation score**	
Satisfactory, n	88
Suboptimal, n	11
Failed, n	2

Table S6. Left and right heart CMR functional assessment by manual analysis.

Manual	***Alive (n = 85)***	***Dead (n = 16)***	***p-value***
**Left heart**			
Left atrial end-diastolic volume, mL	97.6 ± 54.3	104.8 ± 57.5	0.633
Left atrial end-systolic volume, mL	47.5 ± 43.1	63.2 ± 47.8	0.192
Left atrial stroke volume, mL	50.1 ± 18.4	41.5 ± 21.2	0.099
Left atrial ejection fraction, %	55.5 ± 13.9	44.2 ± 15.5	**0.004**
Left atrial global longitudinal strain, %	-16.1 ± 9.2	-12.2 ± 9.9	0.125
Left ventricular end-diastolic volume, mL	171 ± 52.1	158.3 ± 59.6	0.383
Left ventricular end-systolic volume, mL	80.1 ± 46.9	76.1 ± 56.8	0.766
Left ventricular stroke volume, mL	91 ± 24.9	82.2 ± 21	0.188
Left ventricular mass, g	137.3 ± 45.8	163.7 ± 51.3	**0.041**
Left ventricular ejection fraction, %	55.4 ± 12.8	56.1 ± 17.5	0.851
Left ventricular peak ejection rate, mL/sec	444 ± 120	475 ± 286	0.473
Left ventricular peak filling rate, mL/sec	470 ± 170	415 ± 183	0.239
Left ventricular cardiac output, mL/min	6,024 ± 1,668	5,774 ± 1,651	0.584
Left ventricular global longitudinal strain, %	-16.9 ± 5.1	-16.4 ± 7.1	0.770
**Right heart**			
Right atrial end-diastolic volume, mL	73.2 ± 31.4	82 ± 44.6	0.355
Right atrial end-systolic volume, mL	41 ± 27.7	53.6 ± 45.8	0.147
Right atrial stroke volume, mL	32.9 ± 12.5	28.3 ± 9.8	0.178
Right atrial ejection fraction, %	47 ± 12.8	41.5 ± 18.1	0.153
Right atrial global longitudinal strain, %	-16.5 ± 6.8	-14.9 ± 11	0.458
Right ventricular end-diastolic volume, mL	78.4 ± 31	80.4 ± 33.7	0.821
Right ventricular end-systolic volume, mL	27 ± 15.5	32.2 ± 27.7	0.285
Right ventricular stroke volume, mL	51.5 ± 20	48.2 ± 17.3	0.537
Right ventricular ejection fraction, %	67 ± 11	63 ± 15	0.208
Right ventricular cardiac output, mL/min	3,428 ± 1,365	3,354 ± 1,212	0.841
Right ventricular global longitudinal strain, %	-29 ± 7.9	-26.7 ± 11.4	0.326

Data are given as mean ± standard deviation.

Table S7. Left and right heart CMR functional assessment by automated analysis.

AI	***Alive (n = 85)***	***Dead (n = 16)***	***p-value***
**Left heart**			
Left atrial end-diastolic volume, mL	102.7 ± 55.8	106.2 ± 59.4	0.824
Left atrial end-systolic volume, mL	48.8 ± 41.9	64 ± 50.7	0.202
Left atrial stroke volume, mL	55.1 ± 21.1	42.2 ± 21.3	**0.027**
Left atrial ejection fraction, %	57.5 ± 14.9	44.6 ± 16.4	**0.002**
Left atrial global longitudinal strain, %	-22.7 ± 11.2	-16.1 ± 9.5	**0.029**
Left ventricular end-diastolic volume, mL	175 ± 53.4	157 ± 58.1	0.228
Left ventricular end-systolic volume, mL	79 ± 45.1	69.8 ± 43	0.456
Left ventricular stroke volume, mL	96.1 ± 26.2	87.3 ± 33	0.241
Left ventricular mass, g	136.9 ± 47.5	164 ± 51.3	**0.041**
Left ventricular ejection fraction, %	56.8 ± 12.1	57.7 ± 15.6	0.792
Left ventricular peak ejection rate, mL/sec	477 ± 124	508 ± 236	0.437
Left ventricular peak filling rate, mL/sec	517 ± 164	494 ± 485	0.732
Left ventricular cardiac output, mL/min	6,372 ± 1,739	6,205 ± 3,032	0.758
Left ventricular global longitudinal strain, %	-18.9 ± 5.3	-16.6 ± 6.3	0.121
**Right heart**			
Right atrial end-diastolic volume, mL	75.3 ± 29.6	82.7 ± 39.3	0.392
Right atrial end-systolic volume, mL	39 ± 23.8	52.4 ± 42.6	0.079
Right atrial stroke volume, mL	37.3 ± 15.9	30.3 ± 12.5	0.101
Right atrial ejection fraction, %	51.5 ± 14.9	42.2 ± 18.1	**0.029**
Right atrial global longitudinal strain, %	-23.6 ± 10.9	-17 ± 9.8	**0.027**
Right ventricular end-diastolic volume, mL	86.2 ± 31.6	83.6 ± 30.6	0.764
Right ventricular end-systolic volume, mL	31.2 ± 16.2	29.2 ± 15.7	0.642
Right ventricular stroke volume, mL	54.9 ± 19.5	54.4 ± 22	0.921
Right ventricular ejection fraction, %	64.9 ± 9.5	665.5 ± 11.5	0.813
Right ventricular cardiac output, mL/min	3,649 ± 1,326	3,768 ± 1,527	0.749
Right ventricular global longitudinal strain, %	-29.1 ± 6.9	-29.3 ± 12.6	0.942

Data are given as mean ± standard deviation.

Table S8—Univariable and stepwise multivariable Cox regression analysis of all four chambers by manual examination.

Parameter	***b***	***SE***	***Wald***	***p*-value**	***HR***	***95% CI***
**Univariable Cox regression**						
Left atrial end-diastolic volume, mL	0.00	0.00	0.21	0.646	1.00	0.9942 to 1.0094
Left atrial end-systolic volume, mL	0.00	0.00	1.71	0.191	1.00	0.9976 to 1.0121
Left atrial stroke volume, mL	-0.03	0.02	3.06	0.081	0.97	0.9443 to 1.0033
Left atrial ejection fraction, %	-0.04	0.01	8.33	**0.004**	0.96	0.9322 to 0.9867
Left atrial global longitudinal strain, %	0.05	0.03	2.60	0.107	1.05	0.9894 to 1.1158
Left ventricular end-diastolic volume, mL	0.00	0.01	0.72	0.395	1.00	0.9847 to 1.0061
Left ventricular end-systolic volume, mL	0.00	0.01	0.05	0.825	1.00	0.9880 to 1.0097
Left ventricular stroke volume, mL	-0.02	0.01	1.98	0.159	0.98	0.9633 to 1.0061
Left ventricular mass, g	0.01	0.00	3.95	**0.047**	1.01	1.0001 to 1.0175
Left ventricular ejection fraction, %	0.00	0.02	0.00	0.976	1.00	0.9642 to 1.0383
Left ventricular global longitudinal strain, %	0.02	0.05	0.17	0.684	1.02	0.9308 to 1.1156
Right atrial end-diastolic volume, mL	0.01	0.01	0.88	0.348	1.01	0.9934 to 1.0190
Right atrial end-systolic volume, mL	0.01	0.01	2.23	0.135	1.01	0.9973 to 1.0204
Right atrial stroke volume, mL	-0.03	0.00	1.97	0.161	0.96	0.9238 to 1.0132

Right atrial ejection fraction, %	-0.03	0.02	2.39	0.122	0.97	0.9409 to 1.0072
Right atrial global longitudinal strain, %	0.03	0.04	0.80	0.370	1.03	0.9617 to 1.1106
Right ventricular end-diastolic volume, mL	0.00	0.01	0.02	0.890	1.00	0.9858 to 1.0166
Right ventricular end-systolic volume, mL	0.01	0.01	1.12	0.290	1.01	0.9899 to 1.0345
Right ventricular stroke volume, mL	-0.01	0.01	0.51	0.476	0.99	0.9647 to 1.0169
Right ventricular ejection fraction, %	-0.03	0.02	1.82	0.178	0.98	0.9400 to 1.0115
Right ventricular global longitudinal strain, %	0.03	0.03	1.15	0.283	1.03	0.9761 to 1.0863
**Multivariable Cox regression**						
Left atrial ejection fraction, %	-0.04	0.01	8.33	**0.003**	0.96	0.9322 to 0.9867

Table S9—Univariable and stepwise multivariable Cox regression analysis of all four chambers by automated analysis.

Covariable	***b***	***SE***	***Wald***	***p*-value**	***HR***	***95% CI***
**Univariable Cox regression**						
Left atrial end-diastolic volume, mL	0.00	0.00	0.04	0.839	1.00	0.9928 to 1.0089
Left atrial end-systolic volume, mL	0.00	0.00	1.65	0.199	1.01	0.9974 to 1.0127
Left atrial stroke volume, mL	-0.04	0.02	5.66	**0.017**	0.96	0.9361 to 0.9937
Left atrial ejection fraction, %	-0.05	0.01	9.69	**0.002**	0.96	0.9286 to 0.9833
Left atrial global longitudinal strain, %	0.07	0.03	5.66	**0.017**	1.07	1.0115 to 1.1261
Left ventricular end-diastolic volume, mL	-0.01	0.01	1.39	0.238	0.99	0.9824 to 1.0044
Left ventricular end-systolic volume, mL	0.00	0.01	0.44	0.506	1.00	0.9825 to 1.0088
Left ventricular stroke volume, mL	-0.01	0.01	1.63	0.202	0.99	0.9663 to 1.0073
Left ventricular mass, g	0.01	0.00	4.00	**0.046**	1.01	1.0002 to 1.0158
Left ventricular ejection fraction, %	0.00	0.02	0.02	0.899	1.00	0.9630 to 1.0438
Left ventricular global longitudinal strain, %	0.07	0.04	2.70	0.100	1.07	0.9868 to 1.1640
Right atrial end-diastolic volume, mL	0.01	0.01	0.72	0.396	1.01	0.9919 to 1.0207
Right atrial end-systolic volume, mL	0.01	0.01	3.31	0.069	1.01	0.9991 to 1.0246
Right atrial stroke volume, mL	-0.04	0.02	3.19	0.074	0.96	0.9162 to 1.0041

Right atrial ejection fraction, %	-0.04	0.02	5.68	**0.021**	0.96	0.9310 to 0.9931
Right atrial global longitudinal strain, %	0.07	0.03	6.52	**0.011**	1.07	1.0159 to 1.1277
Right ventricular end-diastolic volume, mL	0.00	0.01	0.11	0.740	1.00	0.9817 to 1.0132
Right ventricular end-systolic volume, mL	-0.01	0.02	0.18	0.673	0.99	0.9609 to 1.0261
Right ventricular stroke volume, mL	0.00	0.01	0.04	0.852	1.00	0.9732 to 1.0226
Right ventricular ejection fraction, %	-0.03	0.02	1.82	0.178	0.98	0.9400 to 1.0115
Right ventricular global longitudinal strain, %	0.03	0.03	1.15	0.283	1.03	0.9761 to 1.0863
**Multivariable Cox regression**						
Left atrial ejection fraction, %	-0.05	0.01	9.71	**< 0.001**	0.96	0.9289 to 0.9837

Supplementary Videos and Figures

Video S1—examples of quality control assessment results of the validation cohort: (a) Satisfactory, (b) suboptimal, and (c) failure categorisations.

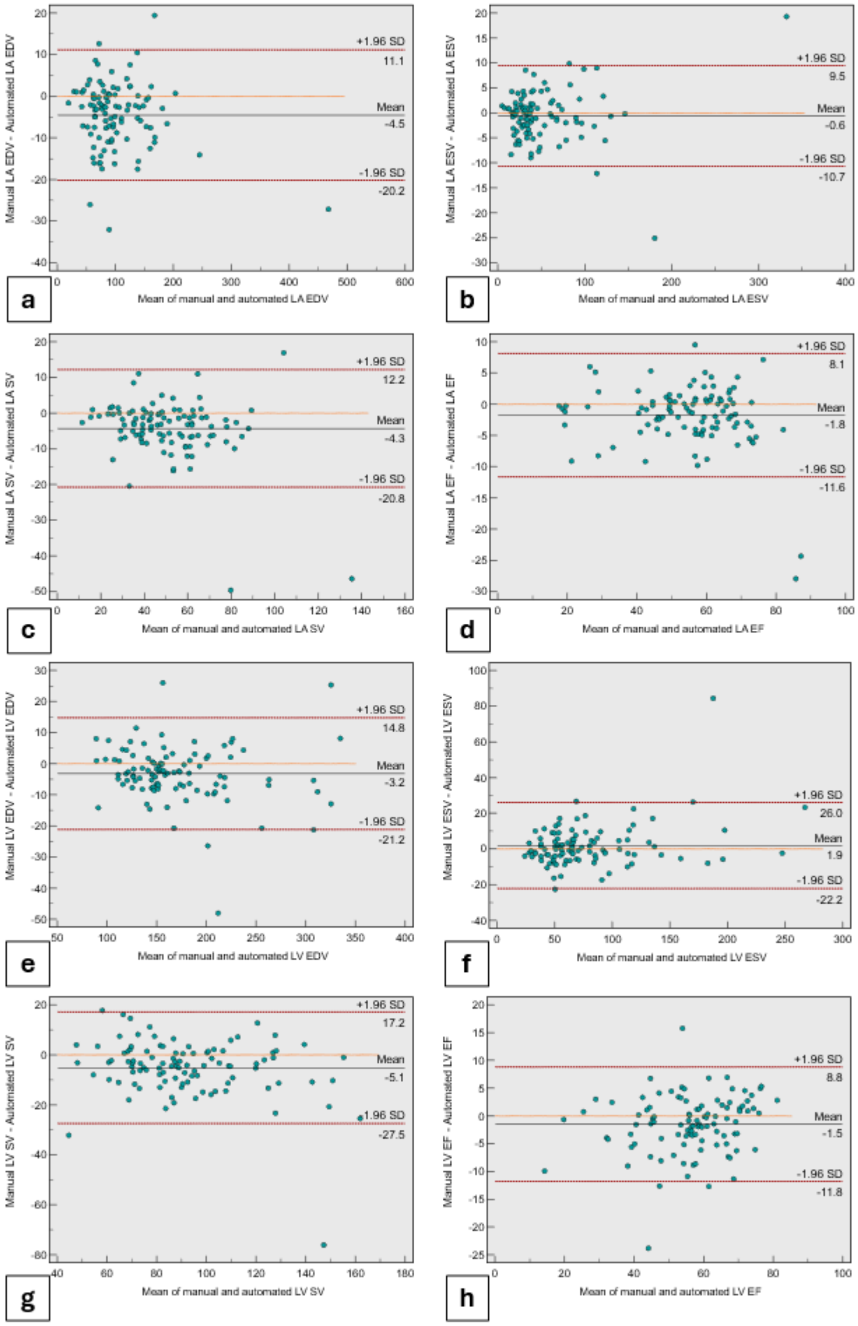

Fig. S1. Bland-Altman plots demonstrating the degree of agreement between manual segmentation and AI four-chamber analysis of the left atrium and left ventricle. (**a**) *LA EDV* left atrial end-diastolic volume. (**b**) *LA ESV* left atrial end-systolic volume. (**c**) *LA SV* left atrial systolic

volume. (**d**) *LA EF* left atrial ejection fraction. (**e**) *LV EDV* left ventricular end-diastolic volume. (**f**) *LV ESV* left ventricular end-systolic volume. (**g**) *LV SV* left ventricular systolic volume. (**h**) *LV EF* left ventricular ejection fraction.

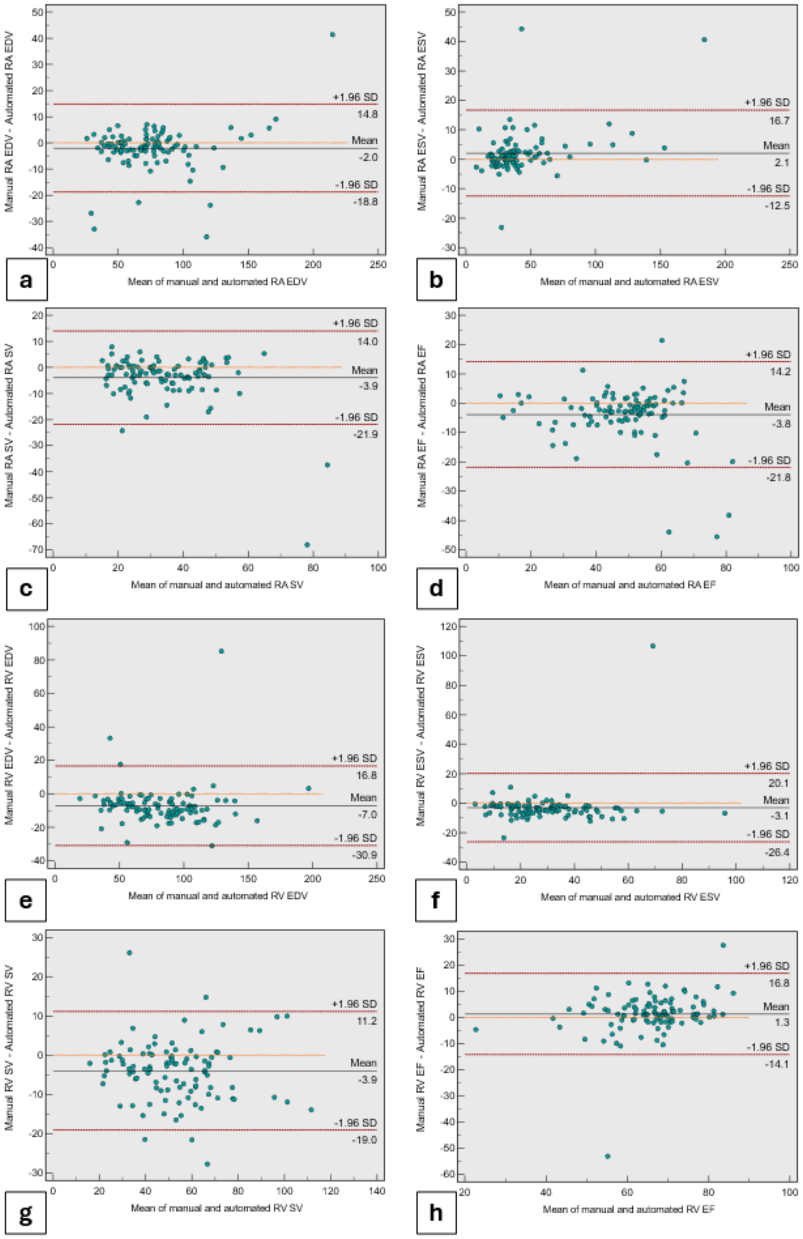

Fig. S2. Bland-Altman plots demonstrating the degree of agreement between manual segmentation and AI four-chamber analysis of the right atrium (a, b, c, d) and right ventricle (e, f, g, h). (**a**) *RA EDV* right atrial end-diastolic volume. (**b**) *RA ESV* right atrial end-systolic volume. (**c**) *RA SV* right atrial systolic volume. (**d**) *RA EF* right atrial ejection fraction. (**e**) *RV EDV* right ventricular end-diastolic volume. (**f**) *RV ESV* right ventricular end-systolic volume. (**g**) *RV SV* right ventricular systolic volume. (**h**) *RV EF* right ventricular ejection fraction.

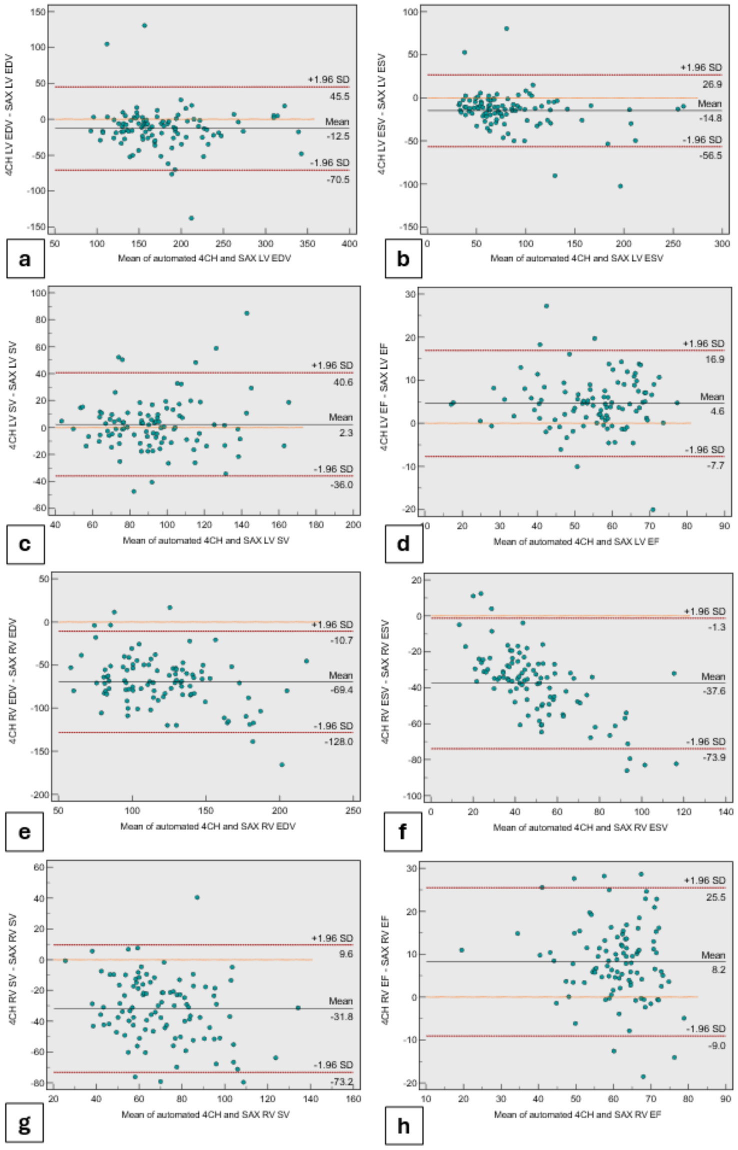

Fig. S3. Bland-Altman plots demonstrating the degree of agreement between AI-generated four-chamber and short-axis segmentations. (**a**) *LV EDV* left ventricular end-diastolic volume. (**b**) *LV ESV* left ventricular end-systolic volume. (**c**) *LV SV* left ventricular systolic volume. (**d**) *LV EF* left ventricular ejection fraction. (**e**) *RV EDV* right ventricular end-diastolic volume. (**f**) *RV ESV* right ventricular end-systolic volume. (**g**) *RV SV* right ventricular stroke volume. (**h**) *RV EF* right ventricular ejection fraction. *4CH* four chambers, *SAX* short-axis.

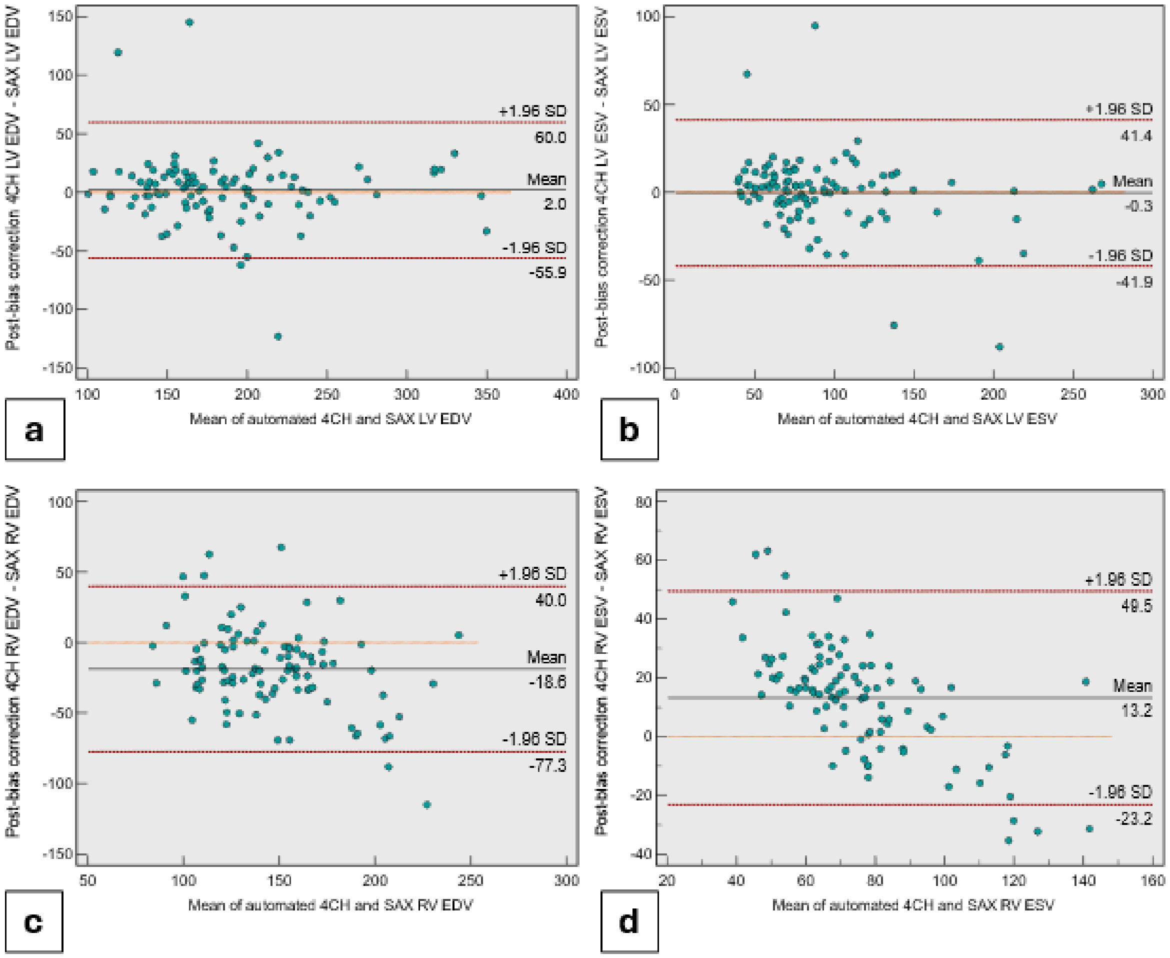

Fig. S4. Bland-Altman plots demonstrating the degree of agreement between AI-generated four-chamber and short-axis segmentations after applying the correction factor (LV=14.5623 mL; RV=50.7676 mL). (**a**) *LV EDV* left ventricular end-diastolic volume. (**b**) *LV ESV* left ventricular end-systolic volume. (**c**) *RV EDV* right ventricular end-diastolic volume. (**d**) *RV ESV* right ventricular end-systolic volume. *4CH* four chambers, *SAX* short-axis.
