# Supplementary figures and images for: Development and validation of AI-derived segmentation of four-chamber cine cardiac magnetic resonance
Source: Eur Radiol Exp. 2024 Jul 12;8:77. doi: 10.1186/s41747-024-00477-7 (PMC11239622; doi:10.1186/s41747-024-00477-7)

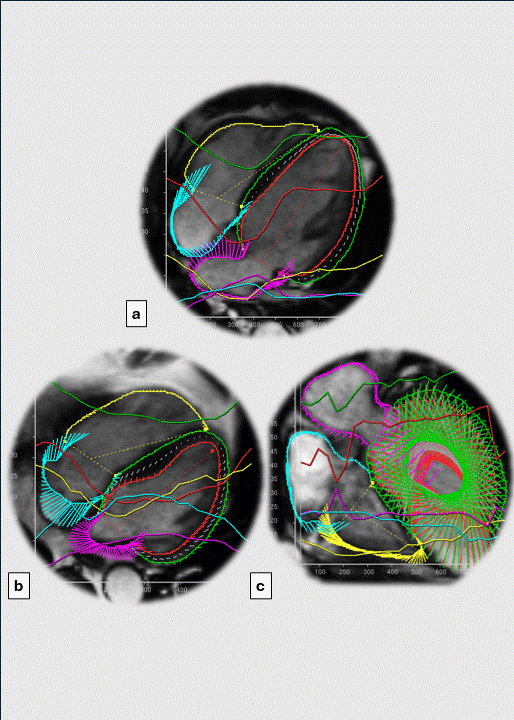

Supplement: Supplementary file 2 — Supplementary Material 2. Examples of quality control assessment results of the validation cohort: (a) Satisfactory, (b) suboptimal, and (c) failure categorisations. [file 41747_2024_477_MOESM2_ESM.gif]
